# Assessing the Preanalytical Variability of Plasma and Cerebrospinal Fluid Processing and Its Effects on Inflammation-Related Protein Biomarkers

**DOI:** 10.1016/j.mcpro.2021.100157

**Published:** 2021-09-29

**Authors:** Jesse Huang, Mohsen Khademi, Örjan Lindhe, Gunn Jönsson, Fredrik Piehl, Tomas Olsson, Ingrid Kockum

**Affiliations:** 1 Neuroimmunology Unit, Department of Clinical Neuroscience, Center of Molecular Medicine, Karolinska University Hospital, Karolinska Institutet, Stockholm, Sweden; 2 Olink Proteomics AB, Uppsala Science Park, Uppsala, Sweden

**Keywords:** blood, CSF, plasma, proximity extension assay, Gompertz function, multiple sclerosis, AXIN-1, axis inhibitor 1, CD40L, cluster differentiation 40 ligand, CNS, central nervous system, CSF, cerebrospinal fluid, CXCL, C-X-C motif chemokine, IL, interleukin, MS, multiple sclerosis, PEA, proximity extension assay, RT, room temperature, SIRT2, sirtuin 2

## Abstract

Proteomics studies are important for the discovery of new biomarkers as clinical tools for diagnosis and disease monitoring. However, preanalytical variations caused by differences in sample handling protocol pose challenges for assessing biomarker reliability and comparability between studies. The purpose of this study was to examine the effects of delayed centrifuging on measured protein levels in plasma and cerebrospinal fluid (CSF). Blood from healthy individuals and patients with multiple sclerosis along with CSF from patients with suspected neurological disorders were left at room temperature for different periods (blood: 1, 24, 48, 72 h; CSF: 1 and 6 h) prior to centrifuging. Ninety-one inflammation-related proteins were analyzed using a proximity extension assay, a high-sensitivity multiplex immunoassay. Additional metabolic and neurology-related markers were also investigated in CSF. In summary, many proteins, particularly in plasma, had increased levels with longer delays in processing likely due in part to intracellular leakage. Levels of caspase 8, interleukin 8, interleukin 18, sirtuin 2, and sulfotransferase 1A1 increased 2-fold to 10-fold in plasma after 24 h at room temperature. Similarly, levels of cathepsin H, ectonucleoside triphosphate diphosphohydrolase 5, and WW domain containing E3 ubiquitin protein ligase 2 differentiated in CSF with <6 h delay in processing. However, the rate of change for many proteins was relatively consistent; therefore, we were able to characterize biomarkers for detecting sample handling variability. Our findings highlight the importance of timely and consistent sample collection and the need for increased awareness of protein susceptibility to sample handling bias. In addition, suggested biomarkers may be used in certain situations to detect and correct for preanalytical variation in future studies.

## Highlights

• Several blood and cerebrospinal fluid proteins are affected by sample handling.
• Plasma protein levels increased with longer centrifugation delay from hemolysis.
• Certain proteins may assess sample handling variability and predict delay time.

## Graphical Abstract

**Figure:**
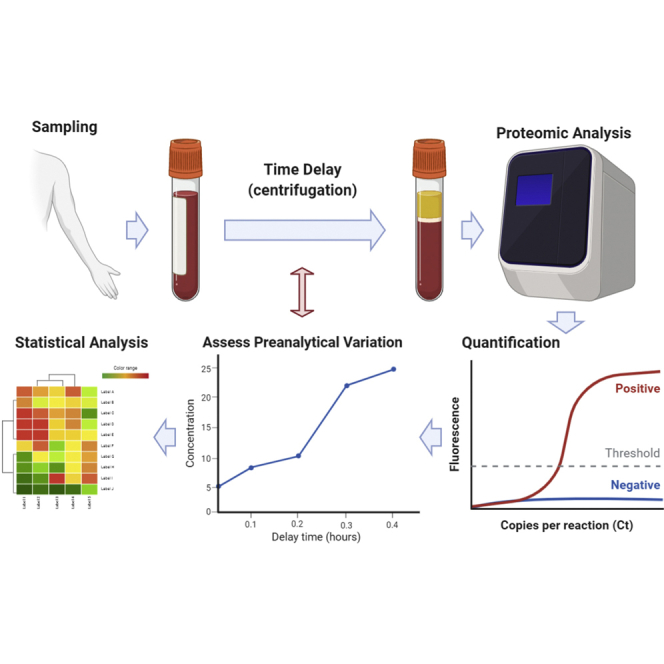

## In Brief

We assessed the effects of delayed sample handling on a panel of 92 inflammation-related proteins in the blood and cerebrospinal fluid. Plasma protein levels were measured at delayed centrifugation times of 1, 24, 48, and 72 h corresponding with common postal-transit delays, and changes in relative concentration were modeled and validated using an external dataset. Several proteins were selected as markers of assessing sample handling variability for application in future studies.

Molecular biomarkers play an important role in the risk assessment, diagnosis, and monitoring of a wide range of diseases (1, 2), and the need for more accurate and reliable clinical tools has instigated the development of high-sensitivity technologies for exploratory proteomics (3, 4). This has allowed recent research into “trace” proteins in the blood, which remains the primary source for biomarker investigation because of its accessibility and role as a biological “sink” for many physiological processes and disorders throughout the body. However, the complex composition of blood, particularly the diverse cellular proteomes that are susceptible to contaminating blood-related media, increases the risk for sample handling bias further amplified by high-sensitivity technologies (2, 5). These challenges are common in multicenter studies where sample processing can be delayed, often inconsistently, because of site-specific logistical restrictions. This increases opportunities for protein degradation as a result of persistent enzyme activity and leakage of intracellular components because of hemolysis (5, 6, 7, 8).

Although blood remains ideal for clinical applications, the exclusivity of the central nervous system (CNS), as maintained by the blood–brain barrier, can compartmentalize potential markers of disease pathology. Therefore, many neurological diseases of the CNS utilize cerebrospinal fluid (CSF) as the primary source of biomarkers for diagnosing and disease monitoring. However, similar to blood, discrepancies in the handling of CSF samples may result in preanalytical variation of protein measures (8, 9). For example, C-X-C motif chemokine ligand 13, a biomarker for multiple sclerosis (MS) (10), can be affected by the frequency of freeze/thaw cycles (11), whereas β-amyloid and total tau levels used for the diagnosis of Alzheimer's disease can also be influenced by delays in sample processing (9, 12). These problems create difficulties in setting standardized cutoffs, which can limit interstudy comparability when assessing diagnostic efficacy (2, 8, 12).

In this study, we examine the preanalytical effects of handling time prior to sample processing on the levels of inflammation-related proteins in both plasma and CSF using high-sensitivity proximity extension technology (3, 13). Our focus here is toward longer delays of 24, 48, and 72 h, although we have also assessed data with shorter delays (3 and 8 h). This time frame is a common condition with large population-based or national registry–based cohorts, particularly those for genetic studies where blood may be collected in collaboration with multiple clinics and posted (1–3 days) to a single processing center. Although not ideal for proteomic research, the vast resources and data typically available from these studies justify evaluating the stability of certain proteins and potential applications for correcting such transit time. Our findings suggest many proteins, particularly in plasma, are severely influenced by preprocessing time. However, changes are predictable and therefore we have suggested models for correcting discrepancies in protein levels because of sample handling, for the purpose of limiting false positives and reducing cross-study variation.

## Experimental Procedures

### Experimental Design

#### Sample Collection and Processing

Blood was collected from three healthy individuals and three patients with MS in 10 ml Vacutainer tubes containing EDTA anticoagulant (Becton Dickinson) as part of the standard routine for the STOPMS-II cohort (approved by Karolinska Regional Ethical Board: 2009/2107-31/2), locally collected at the Karolinska University Hospital (10). Participants provide written consent to participate in the study, which is carried out in accordance with Declaration of Helsinki. Summary details for each subject are given in supplemental Table S1. Blood and CSF samples were taken at a neurology clinic and processed at a separate research laboratory, which is a common setup. Hence, baseline samples were handled within 1 h. Samples were left at room temperature (RT, ∼22 °C) for <1 (baseline), 24, 48, and 72 h prior to processing (centrifuge 1700*g* at 15 min at RT, storage −80 °C). Similarly, CSF was also collected from three patients with other neurological diseases or suspected of MS using a 15 ml size polypropylene DNA-free, DNase-free, RNase-free tube, nonpyrogenic, and noncytotoxic (Sarstedt). Samples were left at RT for <1 (baseline) and 6 h. Two samples, one intact (“whole” CSF) and the other centrifuged (cell-free CSF, 400*g*, 10 min at RT), were compared for both time points. The whole CSF and recovered cell-free CSF were transferred into a 2 ml polypropylene tube (Sarstedt) and stored either at RT or at −80 °C. Both blood and CSF were processed on site within 1 h from collection and stored at −80 °C for less than 2 months before being thawed once, aliquoted, and shipped overnight on dry ice to Olink Proteomics AB for analysis.

#### Proteomic Analysis

Protein concentrations were measured using a proximity extension assay (PEA) (13), a high-throughput immunoassay utilizing paired oligonucleotide antibody-labeled probes. In summary, 1 μl sample was combined with 3 μl incubation probe mix and left incubating overnight on a 96-well plate at 4 °C. An extension mix (96 μl) containing PCR polymerase was added and then transferred to a thermal cycler for extension and preamplification. In the detection phase, 2.8 μl from each well was mixed with 7.2 μl detection mix. Samples were transferred to a 96.96 Dynamic Array Integrated Fluidic Circuit chip with corresponding primers and ran in a Fluidigm BioMark reader using the standard protocol provided by the supplier. Relative protein concentrations were quantified by quantitative PCR as log base-two normalized protein expression values. Measures for each sample were normalized using the internal assay controls of the extension reaction and then further corrected by the triplicate interplate and negative controls as detailed by standard Olink protocol.

The inflammation panel was used for both plasma and CSF analyzing 91 proteins preselected with an emphasis on processes of inflammation and immune activation. Two additional panels (neuroexploratory and metabolism) were also analyzed for CSF to examine the effects of sample handling on metabolic and neurology-related markers in the CNS. Only measures with an overall call rate (*i.e.*, measurable concentration above the limit of detection) of 80% or above were analyzed. However, a few select proteins (plasma monocyte chemotactic protein 3, glial cell–derived neurotrophic factor, interleukin 17C (IL-17C), and IL-10 receptor subunit alpha) with a call rate of 40 to 80% were also examined for changes in detectable presence with increasing processing delay.

#### Statistical Rationale

Data were processed and analyzed with R-3.2.3 (r-project.org). Changes in protein measures between each time point and baseline were analyzed using a paired Student' *t* test. The change in plasma protein levels (P(*t*)) over time (*t*) in comparison to a reference sampling time (*t*_R_) and concentration (P_R_) was modeled using three standard functions: linear {P(*t*) = A(*t* − *t*_R_) + P_R_}, exp {P(*t*) = B(exp(A(*t* − *t*_R_))−1) + P_R_}, and Gompertz {P(*t*) = B ∗ exp(−ln(B/P_R_) ∗ exp(−A(*t* − *t*_R_)))−1}. As the baseline sample (<1 h from collection) was used as a reference, the reference time and value for both linear and exponential models were set at *t*_R_ = 1 and P_R_ = 0. For Gompertz models, protein changes were in reference to the proportion of baseline instead of change from baseline, therefore, *t*_R_ = 1 and P_R_ = 1. Parameters A and B that represent the rate of change and baseline level in the aforementioned formulae were then optimized, and models for each protein were examined for stability and fit. A recommended model was selected depending on whether the rate of change was stable (linear), increasing (exponential), or decreasing (Gompertz) over time. For CSF, only a linear model was used because of limited resolution.

## Results

**Figure 1:**
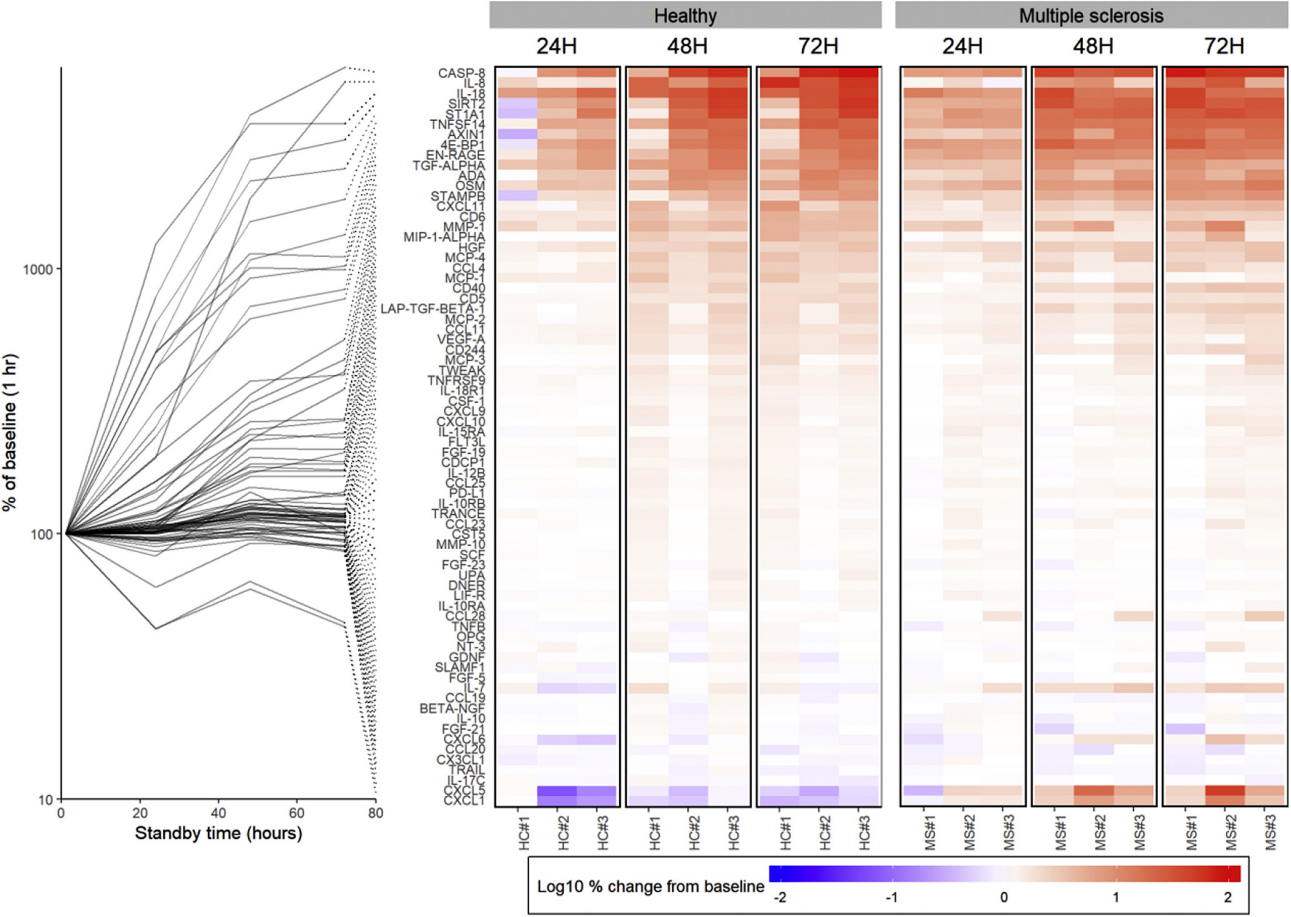
**Effects of delayed centrifugation on protein levels in plasma**. Line plot illustrates the change in plasma protein levels for 72 inflammation-related proteins at 24, 48, and 72 h standby time (room temperature, 22 °C) before sample processing (*i.e.*, centrifuge, −80 °C storage) compared with baseline (<1 h). Heat map illustrates the change in protein level at the same time points compared with baseline for three healthy individuals and relapsing-remitting multiple sclerosis (MS) patients. Proteins are ordered (*top* to *bottom*) from greatest increase to greatest decrease after 72 h.

As illustrated by the time-dependent changes in protein levels from Figure 1, levels for the majority of proteins measured in plasma are positively affected by preprocessing time with caspase 8, IL-8, IL-18, sirtuin 2 (SIRT2), and sulfotransferase 1A1 increasing 2-fold to 10-fold within the first 24 h. The rate of increase tends to be consistent over the observed period and likely due in part to the consequence of cell lysis and leakage of intracellular components. Certain hemolytic factors may be autocatalytic as shown by the increased levels of intracellular axis inhibitor 1 (AXIN-1) and signal-tranducing adaptor molecule binding protein (14) while the resulting accumulation of inflammatory mediators may also cause increased expression of inflammatory cytokines in immune cells, such as IL-8 and macrophage inflammatory protein-1 alpha (7). In addition, the rate also decreases for certain proteins after 48 to 72 h possibly because of convergence between increasing extracellular concentrations and leakage from intracellular compartments; however, in most cases, rates remain stable for the sampled interval. Changes in protein levels are mostly consistent between samples suggesting that the effects of handling can be modeled and predicted (Table 1).

**Table 1:** The modeled effect of delayed sample processing on the levels of sensitive plasma proteins among healthy and MS patients

Protein	CV	Healthy			MS		
	Intra/inter	Model	A	B	Model	A	B
CASP-8	7/22	Gompertz	0.035	84.46	Gompertz	0.02679	135.3
IL-8	6/15	Exponential	0.04381	2.331	Exponential	0.04556	0.6793
IL-18	6/19	Gompertz	0.05898	39.49	Gompertz	0.05122	29.94
SIRT2	8/22	Gompertz	0.0398	39.7	Gompertz	0.03477	47.96
ST1A1	6/25	Gompertz	0.045	28.65	Gompertz	0.03616	40.01
TNFSF14	6/15	Gompertz	0.03654	23.9	Gompertz	0.03872	25.93
AXIN-1	6/19	Gompertz	0.02929	20.23	Gompertz	0.02732	27.23
4E-BP1	6/23	Gompertz	0.05172	12.58	Gompertz	0.04825	22.05
OSM	5/12	Gompertz	0.03353	9.688	Gompertz	0.03813	12.29
CD6	6/23	Exponential	0.01196	2.338	Exponential	0.004072	4.519
HGF	6/16	Gompertz	0.0291	3.261	Gompertz	0.03386	3.274
CXCL6	8/14	Linear	−0.002458	—	Exponential	0.04032	0.07371
CXCL5	7/13	Linear	−0.008607	—	Exponential	0.02917	2.572
CXCL1	6/15	Linear	−0.009023	—	Exponential	0.01642	2.192

The table lists the recommended model and optimized parameters for the top most effected proteins (additional measures are provided in supplemental Table S2). Linear, exponential, or Gompertz curves were used to model changes in plasma protein levels from baseline measure given handling time (hours) prior to sample processing (*i.e.*, centrifuge, −80 °C storage). Predictive models may be used to predict changes in protein levels given a known handling time or (reverse) correct for differences in handling time between cohorts/samples. Models are listed herewith.

Linear {P(*t*) = A(*t* − 1)}.

Exponential {P(*t*) = B(exp(A(*t* − 1))−1)}.

Gompertz {P(*t*) = B ∗ exp(−ln(B) ∗ exp(−A(*t* − 1)))−1}.

Measures of the CV for each protein are provided from Olink product information (www.olink.com). Average within-run precision CV was 6.28, and average between-run CV was 18.83.

Abbreviations: 4E-BP1, 4E-binding protein 1; CASP-8, caspase 8; HGF, hepatocyte growth factor; OSM, oncostatin M; TNFSF14, TNF superfamily member 14.

**Figure 2:**
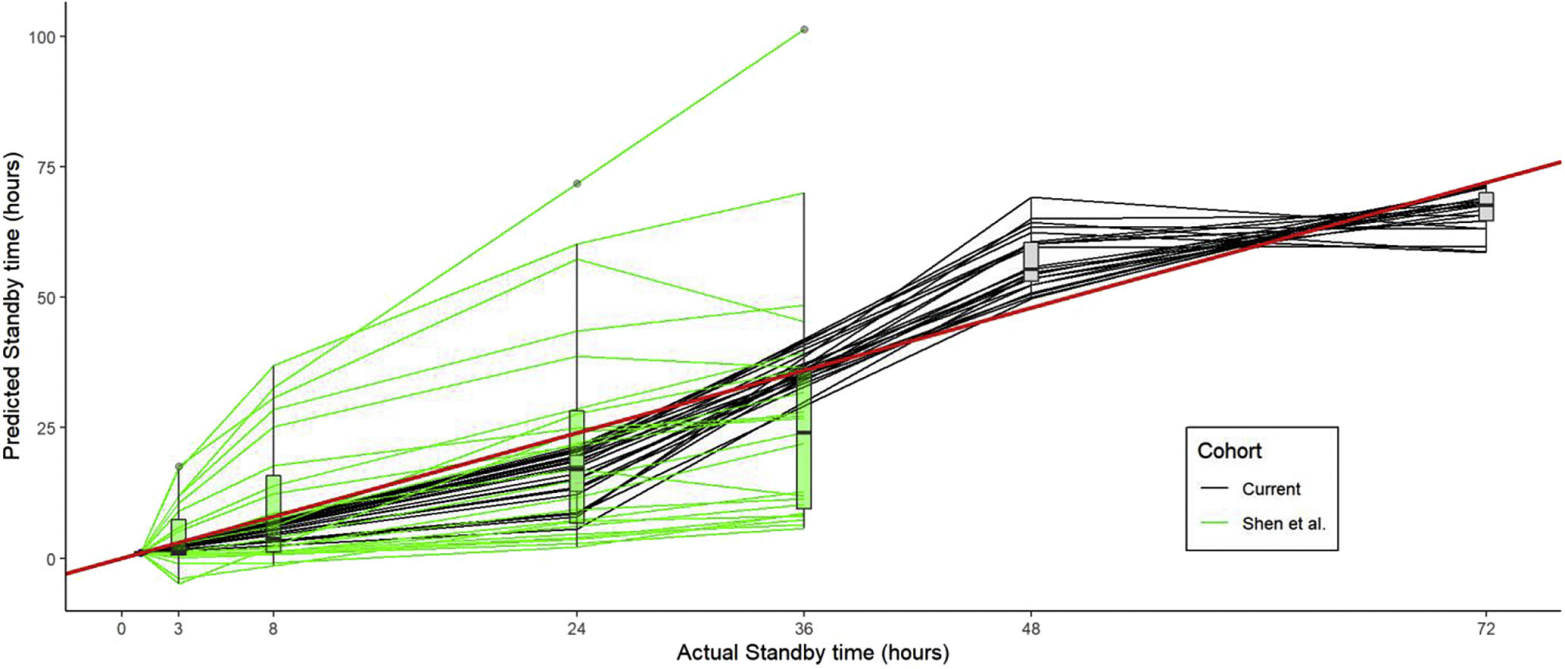
**Assessing predicted sample processing delay time in plasma among both the current cohort and an external validation cohort**. Line plot compares the predicted standby time of samples using the predicted model in Table 1 with the actual standby time for the current cohort (*red*; 24, 48, and 72 h) and Shen *et al.* (*green*; 3, 8, 24, and 36 h) (5). The distribution of the predicted time for all protein measures at each assessed time point is illustrated by box plots. The *red* line is the expected correlation (actual = predicted).

The changes in protein levels detailed in Table 1 were then validated using data from Shen *et al.*, which has sample data from similarly delayed processing but at shorter time points of 3, 8, 24, and 36 h. Time of delay was then predicted based on the changes in protein levels and compared with the actual delay time (Fig. 2). The results showed relative reliability as markers of processing delay, although there was a general trend of overestimation, which could be due to differences in other handling parameters between studies. Best performing markers with the highest precision in the validation cohort includes oncostatin M, hepatocyte growth factor, and cluster of differentiation 6, whereas certain proteins including CXCL6, CXCL5, CXCL1, and IL-7 showed poor predictability and is not recommended for assessing processing delays (Supplemental Table S2).

Because of limited overlap in markers between PEA assay panels, we also investigated proxy markers for preprocessing time in other panels. A cohort of 28 healthy individuals was analyzed for 12 different assay panels, and more than 1000 proteins were examined for their correlation to cluster differentiation 40 ligand (CD40L). Three suggested markers of sample handling for each predetermined panel are listed in Table 2. Soluble CD40L is a platelet-derived cytokine that has been a well-established biomarker of blood sample storage and processing time (5, 7, 15, 16, 17). We also find CD40L to be strongly correlated with levels of AXIN-1, signal-tranducing adaptor molecule binding protein, sulfotransferase 1A1, caspase 8, and SIRT2, which were all primary markers affected by sample handling in this study (Fig. 1).

**Table 2:** Markers for assessing sample handling variability of plasma within each pre-established proximity extension assay panel

Panel name	First marker	Second marker	Third marker
Cardiometabolic	MET (ρ = −0.52; *p* = 0.00573)	TNC (ρ = −0.41; *p* = 0.0336)	FCGR2A (ρ = −0.36; *p* = 0.0667)
Cardiovascular II	CD40L (ρ = 1.00; *p* = NA)	SRC (ρ = 0.80; *p* = 6.44e-07)	HSP27 (ρ = 0.78; *p* = 1.57e-06)
Cardiovascular III	CASP-3 (ρ = 0.88; *p* = 1.79e-09)	JAM-A (ρ = 0.85; *p* = 1.48e-08)	PAI (ρ = 0.66; *p* = 0.000176)
Cell regulation	MAP2K6 (ρ = 0.78; *p* = 2.33e-06)	LRMP (ρ = 0.76; *p* = 6.91e-06)	METAP1D (ρ = 0.74; *p* = 1.65e-05)
Development	CD69 (ρ = 0.86; *p* = 7.69e-09)	SNAP29 (ρ = 0.86; *p* = 9.77e-09)	PPIB (ρ = 0.86; *p* = 1.12e-08)
Immune response	PLXNA4 (ρ = 0.79; *p* = 1.59e-06)	PRDX5 (ρ = 0.78; *p* = 2.16e-06)	EIF4G1 (ρ = 0.76; *p* = 7.8e-06)
Inflammation	AXIN-1 (ρ = 0.74; *p* = 1.76e-05)	STAM-BP (ρ = 0.74; *p* = 1.82e-05)	ST1A1 (ρ = 0.73; *p* = 2.06e-05)
Metabolism	CA13 (ρ = 0.81; *p* = 4.07e-07)	PPP1R2 (ρ = 0.77; *p* = 2.77e-06)	CD2AP (ρ = 0.74; *p* = 9.59e-06)
Neuroexploratory	KIF1BP (ρ = 0.88; *p* = 1.91e-08)	CD63 (ρ = 0.81; *p* = 1.51e-06)	PMVK (ρ = 0.81; *p* = 1.61e-06)
Neurology	MANF (ρ = 0.83; *p* = 2.94e-07)	LAT (ρ = 0.81; *p* = 7.45e-07)	CLEC1B (ρ = 0.79; *p* = 2.35e-06)
Oncology II	EGF (ρ = 0.91; *p* = 6.7e-10)	FADD (ρ = 0.72; *p* = 7.23e-05)	TXLNA (ρ = 0.69; *p* = 0.000176)
Oncology III	GOPC (ρ = 0.81; *p* = 4.41e-07)	CALCOCO1 (ρ = 0.78; *p* = 2.7e-06)	CLIP2 (ρ = 0.77; *p* = 4.19e-06)
Organ damage	BANK1 (ρ = 0.79; *p* = 2.5e-06)	YES1 (ρ = 0.78; *p* = 4.35e-06)	STX8 (ρ = 0.76; *p* = 1.03e-05)

The top three biomarkers for assessing sample handling variability of plasma are listed for each assay panel. Measures were determined and ranked by their predictability of CD40L in a cohort of healthy individuals (n = 28) using a simple linear regression. The Pearson's correlation coefficient (ρ) and probability (*p*) are listed for each protein.

Abbreviations: BANK1, B cell scaffold protein with ankyrin repeats 1; CA13, carbonic anhydrase 13; CALCOCO1,calcium-binding and coiled-coil domain–containing protein 1; CASP-3, caspase 3; CD2AP, CD2-associated protein; CD40L, cluster differentiation 40 ligand; CD63, cluster differentiation 63; CD69, cluster differentiation 69; CLEC1B, C-type lectin domain family 1, member B; CLIP2, CAP-Gly domain–containing linker protein 2; EGF, epidermal growth factor; EIF4G1, eukaryotic translation initiation factor 4 gamma 1; FADD, Fas-associated protein with death domain; FCGR2A, Fc fragment of IgG receptor 2A; GOPC, Golgi-associated PDZ and coiled-coil motif–containing protein; HSP27, heat shock protein 27; JAM-A, junctional adhesion molecule A; KIF1BP, kinesin family member 1-binding protein; LAT, linker for activation of T cells; LRMP, lymphoid-restricted membrane protein; MANF, mesencephalic astrocyte-derived neurotrophic factor; MAP2K6, mitogen-activated protein kinase kinase 6; MET, mesenchymal epithelial transition; METAP1D, methionyl aminopeptidase type 1D; NA, not available; PAI, plaminogen activator inhibitor; PLXNA4, plexin A4; PMVK, phosphomevalonate kinase; PPIB, peptidylprolyl isomerase B; PPP1R2, protein phosphatase inhibitor 2; PRDX5, peroxiredoxin-5; SNAP29, synaptosomal-associated protein 29; SRC, Src family of protein kinases; ST1A1, sulfotransferase 1A1; STAM-BP, signal-tranducing adaptor molecule binding protein; STX8, syntaxin 8; TNC, tenascin C; TXLNA, taxilin alpha; YES1, YES proto-oncogene 1, Src family tyrosine kinase.

**Figure 3:**
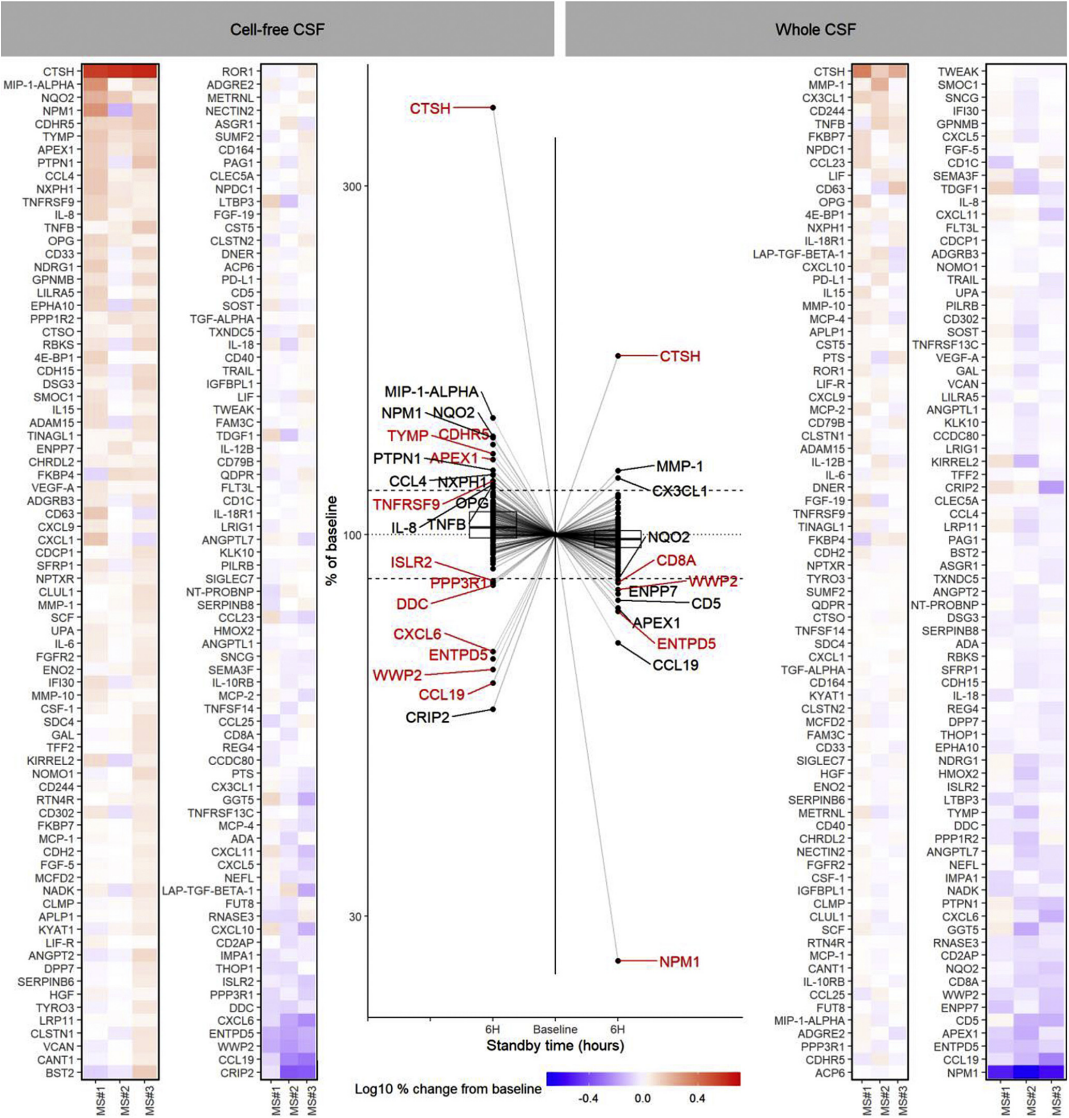
**Effects of delayed centrifugation on protein levels in CSF**. Line plot illustrates the change in CSF protein levels for 156 proteins after 6 h standing time (room temperature, ∼22 °C) before centrifugation (cell-free, CF; *left side*) or no centrifugation (whole, WH; *right side*) compared with baseline (<1 h). Proteins with a change of >15% over 6 h (*dashed lines*) are tagged with those having *p* < 0.10 highlighted *red*. Heat map illustrates the change in protein level for three patients initially suspected with multiple sclerosis (MS). Proteins are ordered from greatest increase (*top* of *left* column) to greatest decrease (*bottom* of *right column*). CSF, cerebrospinal fluid.

To a lesser degree, proteins in CSF were also affected by sample handling as shown in Figure 3. Levels of cathepsin H were notably increased in both cell-free and whole CSF, whereas several proteins, including ectonucleoside triphosphate diphosphohydrolase 5, WW domain-containing protein 2, and CCL19, had decreased levels after just a 6 h delay at RT. Levels of CCL19 and CXCL6 decreased in both CSF as well as plasma. While commonly considered as stable, neurofilament light levels showed a minor decrease at ∼1% per hour. Although findings were relatively consistent between cell-free and whole CSF, cell-free CSF showed a general increase in overall protein level after 6 h, whereas whole CSF showed a neutral or a slight decreasing distribution. This is further evidenced by the markers nucleophosmin 1 and apurinic/apyrimidinic endodeoxyribonuclease 1, and to a lesser degree with NRH dehydrogenase (quinine) 2 and ectonucleotide pyrophosphatase/phosphodiesterase family member 7, which showed a negative correlation with handling time in whole CSF but a positive correlation in cell-free CSF. This difference may be due to the centrifuging process, which may exacerbate protein leakage resulting in the increased presence of intracellular proteins or additional enzymatic activity postcentrifugation although this requires further investigation. As with plasma, the rate of change for several proteins in CSF was relatively consistent between samples and therefore might be appropriately modeled (Table 3).

**Table 3:** Rate of change in CSF protein levels caused by delays in sample processing in cell-free and whole CSF of patients with or suspected of neurological disorders

Protein	CV	R_CF_ (%)	SE_CF_	P_CF_	R_WH_ (%)	SE_WH_	P_WH_
	Intra/inter						
CTSH	5/11	56.91	3.38	0.00107	15.16	5.25	0.0666
ENTPD5	9/11	−6.48	0.38	0.0053	−4.3	0.45	0.0135
WWP2∗	6/15	− 6.93	0.45	0.0066	−3.19	0.63	0.0436
CDHR5∗	5/10	6.56	0.74	0.00933	−0.28	1.3	0.805
CHRDL2∗	6/10	1.76	0.18	0.00974	−0.03	0.47	0.937
TYMP	9/13	5.81	1.14	0.0285	−1.65	1.65	0.415
DDC	5/12	−2.95	0.53	0.0343	−1.67	0.48	0.078
NPTXR	6/12	1.46	0.3	0.0369	0.28	0.5	0.652
APEX1∗	6/14	5.33	1.24	0.0433	−4.11	1.31	0.106
CD2AP	6/8	−1.57	0.72	0.169	−2.43	0.26	0.0129
ADA	5/29	−1.08	1.09	0.422	−1	0.14	0.0205
RNASE3	6/18	−1.4	1.81	0.474	−2.36	0.39	0.03
NPM1∗	4/16	7.13	7.31	0.541	−14.79	0.51	0.00561

Rates of change (R, %) in protein levels per hour of delay in sample processing as determined by the comparison of samples with baseline (<1 h) and 6 h delay at room temperature (22 °C) are given for both cell-free (CF) and whole (WH) CSF. Only proteins with a significant change (*p* < 0.05) within 6 h as determined by standard Student's *t* test are listed. The relative variability in inter/intra runs along with the rate of change for additional proteins, which are provided in supplemental Table S3. Highlighted proteins (∗) showed changes in detectable presence between the two sampled time points; therefore, rate is an approximation.

Abbreviations: ADA, adenosine deaminase; APEX1, DNA-(apurinic or apyrimidinic site) lyase; CD2AP, CD2-associated protein; CDHR5, cadherin-related family member 3; CHRDL2, chordin-like 2; CTSH, cathepsin H; DDC, aromatic-l-amino-acid decarboxylase; ENTPD5, ectonucleoside triphosphate diphosphohydrolase 5; NPM1, nucleophosmin 1; NPTXR, neuronal pentraxin receptor; RNASE3, ribonuclease, RNase A family, 3; TYMP, thymidine phosphorylase; WWP2, WW domain containing E3 ubiquitin protein ligase 2.

## Discussion

Our findings suggest that the levels of many proteins, particularly those in plasma, can be affected by delays in centrifugation, which is supported by previous studies (5, 7, 18). As most proteins affected are of intracellular origin, plasma that had delayed processing is likely contaminated from hemolysis, a common issue in blood sample processing (5, 8). However, certain proteins particularly cytokines such as IL-1RA and oncostatin may be secreted by stimulated immune cells over time as a result of accumulating inflammatory mediators in the extracellular compartment and therefore not directly caused by cell lysis (7, 19, 20), whereas others including CXCL6 are known to degrade by protolytic enzymes (21, 22).

The relative consistency in protein level changes between samples of both healthy and diseased individuals indicates the value of biomarkers for detecting preanalytical variation. By selecting certain proteins as markers of delayed processing, one can detect preanalytical variability by examining sampling parameters such as sampling site, cohort, and periods; or measures of clinical characteristics such as exposures and disease status, which may unknowingly bias study comparisons. The usage of multiple markers can provide confirmation or may be necessary in cases where markers overlap with those of study interest. For each of the pre-established PEA assay panels, we have suggested several markers to aid in the detection of sample handling issues for future studies. As discussed previously, markers were selected based on their correlation to CD40L, a well-established marker of blood storage time and highly sensitive to sample handling variation (5, 15).

When a handling-related bias is suspected, it might be possible to correct protein measures between samples or comparison groups as previously shown with NMR metabolomics data for lipids, amino acids, sugars, and others (23). This can be done by (step 1) approximating the likely difference in preprocessing time using one or more markers of sample handling (*e.g.*, AXIN-1, SIRT2) with the predicted rate of change (Tables 1 and 3) as we have shown with data from Shen *et al.*; and then (step 2) correcting other protein levels by predicting the deviation between the actual level and that expected from the determined time difference in step 1. However, this method only provides a rough approximation and does not factor in many other environmental and sample processing–related variables, which may influence protein measures (24). For example, certain diseases may affect blood cell composition and hemolytic rate (8, 25). Although this could partly explain the inconsistencies between MS patients and controls, the difference in the immune cell composition of MS patients is unlikely to be the primary cause of such discrepancies in protein behavior as the measurable difference in the peripheral inflammatory proteome of MS patients is minimal compared with effects of handling variability (26). Instead, it could be due to their sensitivity against other preanalytical factors as shown by the significant variation of measures like CXCL5 and CXCL1 during external validation. In CSF, minute blood contamination because of factors in sample collection can affect protein measures through direct hemolytic contamination or protein degradation caused by the introduction of enzymes (6, 27, 28). A more conservative method may be to filter phenotype-associated biomarkers by crosscomparing its stability to sample handling through correlation to sample handling markers. This is particularly useful for identifying biomarkers of clinical relevance as proteins need to be stable and reliable enough for clinical application, a setting where sample handling may vary.

With the increase in international and multicenter collaborations accompanied by the usage of high-sensitivity omic technologies, there should be increased awareness of the importance of standardizing protocols for sample handling and the establishment of systematic procedures for documenting and detecting discrepancies. Investigators should pay attention to the nature of their associated markers particularly their stability to handling procedures when interpreting results. This also emphasizes the importance of validating novel biomarkers using separate cohorts with independent sampling handling procedures to ensure the reliability of findings.

## Data Availability

Data are available at the Swedish National Data Service (https://snd.gu.se/en) and upon request to the corresponding author, Ingrid Kockum, Department of Clinical Neuroscience, Karolinska Institutet, Ingrid.kockum@ki.se.

## Supplemental data

This article contains supplemental data.

## Conflict of interest

T. O. has received lecture and/or advisory board honoraria and unrestricted MS research grants from Astrazeneca, Biogen, Novartis, Merck, Roche, Almirall, and Genzyme. F. P. has received research grants from Biogen, Genzyme, Merck KGaA, and Novartis and fees for serving as Chair of DMC in clinical trials with Parexel. Ö. L. is an employee of Olink Proteomics. J. H., M. K., G. J., and I. K. declare no competing interests.
